# Unlocking Antioxidant Potential: Interactions Between Cyanidin-3-Glucoside and *Corbicula fluminea* Protein

**DOI:** 10.3390/biology14101392

**Published:** 2025-10-11

**Authors:** Sifan Guo, Xuemei Liu, Fei Wang, Yong Jiang, Lili Chen, Meilan Yuan, Li Zhao, Chunqing Bai

**Affiliations:** National R&D Branch Center for Freshwater Fish Processing, College of Life Science, Jiangxi Science and Technology Normal University, Nanchang 330013, China; 15903436065@163.com (S.G.); 19570118295@163.com (X.L.); fei_wang0828@163.com (F.W.); 1020130968@jxstnu.edu.cn (Y.J.); 1020140971@jxstnu.edu.cn (L.C.); 1020100975@jxstnu.edu.cn (M.Y.); 1020100962@jxstnu.edu.cn (L.Z.)

**Keywords:** Corbicula fluminea protein, interaction, antioxidant, Cyanidin-3-glucoside

## Abstract

**Simple Summary:**

Oxidative stress is an important inducer of chronic diseases such as cancer and diabetes. Supplementation of exogenous natural antioxidants has become an efficient strategy to alleviate oxidative damage. Polyphenols (e.g., cyanidin-3-O-glucoside and C3G) and aquatic proteins (e.g., *Corbicula fluminea* protein and CFP) are good candidates. Importantly, these two classes of biomolecules are often combined during food processing, leading to interactions that may significantly alter their properties, particularly their antioxidant capacity. Moreover, the extent of their effects are largely dependent on the strength of the polyphenol–protein affinity, which arises from binding mechanisms and is contingent upon the specific polyphenols/proteins, with some systems exhibiting synergistic effects and others demonstrating antagonistic effects. However, information on the interaction between CFP and C3G remains unclear. In this work, the interaction mechanism underlying the physical mixing of C3G and CFP was analyzed in terms of multi-spectroscopy, thermodynamic analysis, and particle properties. The synergistic antioxidant effect of C3G with CFP was also evaluated. The results show that C3G tended to spontaneously bind to CFP, altered its conformation, and formed a compact non-covalent complex. Although the presence of C3G resulted in enhanced antioxidant capacity, the impact was not significant at high C3G levels. What is more, C3G displayed an antagonistic effect when combined with CFP.

**Abstract:**

Corbicula fluminea protein (CFP) and cyanidin-3-O-glucoside (C3G) are natural nutrient fortifiers. During consumption or processing, they may interact with each other, inducing alternations in their structural and functional properties. However, nothing was known about the mechanism of their interaction and their synergistic antioxidant effect. In this research, C3G was physically mixed with CFP to simulate practical scenarios. The impact of the presence of C3G on the multispectral characteristics, antioxidant activity, and particle properties of CFP was examined and compared to chemically fabricated C3G-CFP covalent conjugates. The results indicate that C3G tended to spontaneously bind to CFP and formed compact non-covalent complex, with hydrophobic forces predominantly governing the interaction. This binding resulted in the statically quenched intrinsic fluorescence of CFP, accompanied by a dynamic model. Moreover, C3G preferentially induced Trp residue in CFP exposed to a more polar microenvironment, yet it exerted nearly no effects on CFP when analyzed using ultraviolet–visible (UV-Vis) spectroscopy and synchronous fluorescence spectroscopy (SFS). Additionally, although the formed non-covalent complex demonstrated strengthened antioxidant capacity, C3G displayed an antagonistic effect with CFP, whereas lower C3G concentrations led to synergistic effects in covalent conjugates. These findings provide new insights into the effective application of C3G and CFP as nutritional antioxidants.

## 1. Introduction

In recent years, oxidative stress has been increasingly recognized as a significant contributor to the pathogenesis of chronic diseases, including cancer, diabetes, and cardiovascular disorders, thereby posing a substantial challenge to global public health [1,2]. Although endogenous antioxidant systems offer fundamental protection, their capacity in the human body is inherently limited. Consequently, the supplementation of exogenous antioxidants, particularly those derived from natural sources, has emerged as a critical strategy for mitigating oxidative damage and associated health risks [3,4]. Among diverse natural antioxidants, polyphenolic compounds and proteins are particularly notable. Their excellent antioxidant activity, biocompatibility, and health-promoting properties establish them as primary candidates for exogenous antioxidant supplementation.

Polyphenols are a major class of bioactive compounds abundantly present in plant-based foods, such as fruits (e.g., berries and apples), vegetables (e.g., spinach and onions), cereals, tea, coffee, and red wine [5]. With one or more hydroxyl groups in their molecular makeup, they exert superior antioxidant activity in addition to significantly contributing to the functional properties (antioxidant, anti-inflammatory, antiaging, and anticarcinogenic effects) and sensory qualities (e.g., color, astringency, and flavor) of foods. Proteins also demonstrate a notable antioxidant capacity by delaying lipid oxidation and safeguarding human tissues and organs from free radical damage, while containing essential amino acids [6].

Importantly, these two classes of biomolecules are often combined during food processing, leading to interactions that can significantly alter their properties, particularly their antioxidant capacity. Moreover, the nature and extent of these effects are largely determined by the strength of the polyphenol–protein affinity, which arises from binding mechanisms, such as non-covalent interactions (e.g., hydrogen bonding, hydrophobic interactions, and electrostatic forces) and covalent bonds [7]. Generally, non-covalent bindings are reversible and dynamically influence molecular conformation, whereas covalent binding typically occurs under specific conditions and results in the formed complex exhibiting higher stability, with significant effects on structural and functional properties [8]. In addition, it was found that the binding of polyphenols may affect the antioxidant capacity of proteins by modulating molecular stability, exposing active sites, or facilitating electron transfer pathways [7]. Furthermore, the magnitude of the effect is contingent upon the types/concentrations of polyphenols/proteins and the interaction types, with some systems exhibiting synergistic effects while others demonstrate antagonistic effects [9].

To explore the interactions between polyphenols and proteins and their implications as natural antioxidants, two representative molecules: Cyanidin-3-O-glucoside (C3G) (a anthocyanin) and *Corbicula fluminea* protein (CFP) (an aquatic protein) were selected as models in this study. C3G is a prominent polyphenolic compound commonly found in black rice, purple sweet potatoes, grapes, and blueberries [10]. As illustrated in the graphic abstract, the molecular structural formula of C3G is C_21_H_21_O_11_. Its five phenolic hydroxyl groups in its structure endow it with potent antioxidant activity, as well as anti-inflammatory, anti-cancer, and cardiovascular protective effects, making it a typical and well-studied polyphenol for antioxidant-related research [11]. CFP is a protein extract derived from the freshwater clam, which is rich in essential amino acids and active groups, such as sulfhydryl and hydroxyl groups, and demonstrates unique capabilities in metal ion chelation and free radical scavenging [12]. As an aquatic protein, CFP represents a large but understudied category of proteins, distinct from the plant- or mammalian-derived proteins commonly used in the existing research.

In recent years, the interactions between polyphenols and proteins have become a key focus in food science and medicine. However, existing studies predominantly concentrate on plant-derived polyphenols interacting with plant or mammalian proteins, leaving critical gaps: the interaction mechanisms between polyphenols and aquatic proteins (e.g., CFP) remain insufficiently understood, and the impact of such interactions on antioxidant activities has not been systematically explored. Additionally, current research on CFP primarily focuses on optimizing extraction processes for individual components or evaluating its standalone functional properties, with no systematic investigation into its synergistic effects with bioactive molecules like polyphenols (e.g., C3G). Given that aquatic proteins are valuable nutritional resources and anthocyanins (e.g., C3G) are widely used in functional foods, clarifying the interaction between C3G and CFP and their potential synergistic antioxidant effects could provide novel insights for developing combined antioxidant strategies to prevent chronic diseases.

Against this backdrop, C3G and CFP were selected as polyphenol and aquatic protein models, respectively. The molecular interaction mechanisms between them and their synergistic antioxidant effects were assessed in detail, thereby laying a scientific foundation for the application of combined anthocyanins and aquatic proteins in chronic disease prevention.

## 2. Materials and Methods

### 2.1. Materials

Cyanidin-3-O-glucoside (purity ≥ 98%) was purchased from Shanghai Yuanye Biotechnology Co., Ltd. (Shanghai, China). CFP was extracted from frozen river clam provided by Suzhou Taijin Food Co., Ltd (Suzhou, China). All other chemical reagents used were of analytical grade.

### 2.2. Preparation of CFP

The peptides were prepared according to the method reported by Wang et al. [12]. The frozen clam meat was thawed, washed, drained of excess water, and air-dried for 4 h. After being ground by the meat grinder, the meat was mixed with distilled water at a weight ratio of 1:7, the pH was adjusted to 8.5, and 3 wt% alkaline protease was added. After hydrolysis at 55 °C for 3 h, the mixture was subjected to centrifugation at 5000 r/min for 20 min, and the supernatant was freeze-dried to powder, which was stored at −20 °C for later use. The protein content of the CFP obtained was 96.5% (dry basis), which was measured by the Kjeldahl method [13].

### 2.3. Preparation of C3G-CFP Complexes

C3G-CFP non-covalent complexes and covalent conjugates were prepared according to Wang’s report [12], with slight modification. In brief, CFP (1 mg/mL) and C3G (1 mmol/L) solutions were pre-prepared by individually dissolving them in 10 mmol/L PBS buffer solution (pH 7.0). After dilution to appropriate concentrations, the solutions were mixed, the pH values were adjusted, and the mixture was reacted in the dark. For C3G-CFP non-covalent complexes, the pH was 7.0 and the reaction time was 2 h, while those for the C3G-CFP covalent conjugates were 9.0 and 24 h, respectively. Due to variations in the optimal concentrations for different analyses, the specific concentrations of C3G and CFP are provided in the sections describing their respective methods.

### 2.4. Determination of Particle Size and Zeta Potential

The particle size and zeta potential of free CFP (0.2 mg/mL), C3G-CFP non-covalent complexes, and C3G-CFP conjugates (the concentration of C3G was 35.87 × 10^−6^ mol/L) were determined using a particle size and zeta potential analyzer [14]. Each group of tests was repeated at least three times.

### 2.5. Determination of Endogenous Fluorescence Spectrum

The endogenous fluorescence spectrum of samples was determined referring from Huizenga et al. [15]. The C3G-CFP non-covalent complexes and conjugates were prepared by controlling the concentration of CFP at 0.2 mg/mL and the concentration of C3G within a range of 0–35.87 × 10^−6^ mol/L. After fully oscillating and reacting for 2 h at different temperatures (298 K, 304 K, 310 K), the emission fluorescence spectrum (300–500 nm) of the samples, excited at 280 nm, was scanned and recorded on a F-2700 Fluorescence Spectrophotometer (Hitachi, Ltd., Tokyo, Japan).

### 2.6. Synchronous Fluorescence Spectrum

The synchronous fluorescence spectrum of the non-covalent complexes (0–35.87 × 10^−6^ mol/L of C3G) incubated at 298 K was determined by setting the excitation and emission slit widths at 5 nm, Δλ at 15 nm and 60 nm intervals, and the excitation wavelength of 300–450 nm [16].

### 2.7. Ultraviolet-Visible (UV-Vis) Absorption Spectrum

The UV-vis absorption spectra of the samples used in Section 2.6 were determined on a UV-vis spectrophotometer (U-T6A, Yipu Co., Ltd., Beijing, China), and the absorption spectrum was recorded in the range of 200 to 400 nm [17].

### 2.8. Antioxidant Properties Determination

The antioxidant capacity of the C3G-CFP complexes was evaluated using three methods (ABTS, DPPH and FRAP). It should be noted that the concentration of CFP was kept at 0.1 mg/mL, and the final concentrations of C3G were 0, 2.38, 6.52, 10.00, 12.96, and 15.52 mg/L, respectively. The antioxidant capacity of a free C3G solution at the same concentration was also determined for comparison. Each sample was measured in triplicate to ensure accuracy.

#### 2.8.1. ABTS Method

The ABTS free radical scavenging ability of the samples was conducted according to the method described by Wang et al. [18]. ABTS^+^ working solution was prepared by mixing 7 mM of ABTS with 2.45 mmol/L K_2_S_2_O_8_ solution. This mixture was reacted in the dark for 12–16 h, followed by dilution with PBS (pH 7.0, 10 mM) until its absorbance reached 0.7 ± 0.02 (at 734 nm). Then, 200 μL of sample was added to 3 mL of ABTS^+^ working solution, incubated at room temperature in the dark for 6 min, and then determined at 734 nm. The results were expressed as the Trolox equivalent antioxidant capacity (TEAC, mg/L). The absorbance of the control was measured under the same conditions, using PBS buffer as a substitute for the sample in the ABTS reaction.

#### 2.8.2. DPPH Method

A 1,1-diphenyl-2-picrylhydrazyl radical (DPPH) assay was carried out as described in our previous report [18]. First, 300 μL of sample was mixed with 3 mL of DPPH ethanol working solution (0.5 × 10^−4^ mol/L). This mixture was shaken thoroughly and left in the dark for 30 min. The absorbance of the reacted mixture was measured at 519 nm, and the DPPH radical scavenging ability of samples was expressed in TEAC (mg/L). The blank and control samples were also analyzed following the same procedure. For the blank, absolute ethanol was used instead of the sample, while for the control, it replaced the DPPH working reagent.

#### 2.8.3. FRAP Method

A 2,4,6-Tri(2-pyridyl)-s-triazine (TPTZ) working solution was prepared by mixing 10 mM of TPTZ solution, 0.3 M of sodium acetate buffer, and 20 mM of FeCl_3_ aqueous solution at a volume ratio of 1:10:1. Then, 40 μL of sample was added to 4 mL of the TPTZ working solution. The mixture was then transferred to a water bath kept at 37 °C. After 10 min, the absorbance of the sample was determined at 593 nm [19]. The blank and control samples were also analyzed following the same procedure. For the blank, deionized water replaced the sample in the reaction with the TPTZ solution, whereas for the control, it replaced the TPTZ solution in the reaction with the sample.

#### 2.8.4. Definition of Synergistic Effect

To better describe the antioxidant interaction between CFP and C3G, the synergistic effect was identified according to the following equations:

(1) $$\;\mathrm{TSC}\;(\%)\;=\;100\;-\;\frac{\left(100\;-\;\mathrm{ESC}1\right)\;\times \;(100\;-\;\mathrm{ESC}2)}{100}$$

(2) $$SE\;=\;\frac{\mathrm{ESC}}{\mathrm{TSC}}$$

Here, TSC represents the theoretical scavenging activity; ESC, ESC1, and ESC2 represent the scavenging activity of complexes CFP, and C3G, respectively; SE represents the synergistic effect, which occurs when SE > 1.

### 2.9. Statistical Analysis

The data were expressed as the means ± standard deviation (SD) of at least three measurements. Charts were generated using Origin 2024 version. One-way ANOVA with Duncan’s test was performed on IBM SPSS Statistics (version 26) to evaluate statistical significance, with *p* < 0.05 considered significant.

## 3. Results and Discussion

### 3.1. Fluorescence Spectroscopy

The intrinsic fluorescence of proteins is mainly produced by fluorescent amino acids, including tryptophan (Trp), tyrosine (Tyr), and phenylalanine (Phe). Specially, when proteins are excited at 280 nm, Tyr and Trp residues emit intrinsic fluorescence with maximum peaks located at around 313 and 350 nm, respectively. Notably, these spectra are highly sensitive to the microenvironment, making them widely used to identify changes in protein conformation. Such changes may directly affect the protein’s functional properties (e.g., antioxidant activity) and potential biological applications (e.g., stability, bioavailability). In this section, the effects of various concentrations of C3G on the fluorescence spectra of CFP are investigated.

**Figure 1:**
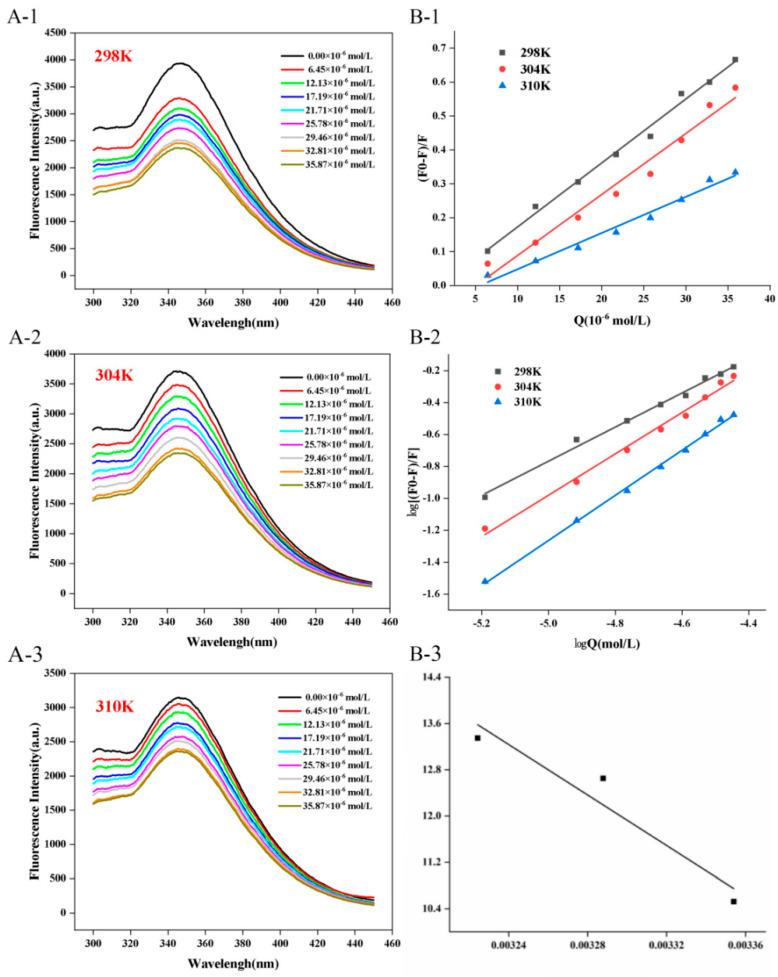
Fluorescence emission spectrum ((**A-1**) 298 K; (**A-2**) 304 K; (**A-3**) 310 K), Stern-Volmer curve (**B-1**), log-log curve (**B-2**), and Van’t Hoff curve (**B-3**) of CFP (0.2 mg/mL) in presence of C3G (0–35.87 × 10^−6^ mol/L) at different temperatures (298 K, 304 K, 310 K).

As illustrated in Figure 1, CFP demonstrated fluorescence absorption ranging from 300 nm to 450 nm when excited at 280 nm, with a pronounced fluorescence emission peak at approximately 346 nm, predominantly attributed to Trp [12]. Upon physical mixing with C3G, a notable decrease in fluorescence intensity was observed, which further diminished with increasing concentrations of C3G. The changes in fluorescence quenching imply that the fluorescence emission of Trp was diminished in the presence of C3G, which may interact with CFP and disrupt the native microenvironment of Trp residues [12]. More C3G may induce stronger disruption in the microenvironment, leading to overall more significantly decreased fluorescence intensity [12,19]. In addition, as the concentration of C3G increased, a slight red shift (2 nm) in the maximum absorption peak of the spectrum was observed. This shift has been found to be associated with changes in the microenvironment polarity of Trp and may indicate conformational alterations in the protein structure [20]. Generally, a red shift signifies that Trp residues are exposed to a more polar microenvironment, whilst a blue shift indicates the microenvironment hydrophobicity around the residue amino acid increases. Consequently, a red shift in the presence of higher amounts of C3G suggests that C3G binding reduces the hydrophobicity or increases the polarity around Trp residues. Furthermore, the increased polarity may partially contribute to the enhanced antioxidant activity of CFP, as stated in Section 3.7.

Furthermore, temperature was found to significantly influence the quenching process, with higher temperatures enhancing the quenching effect. For instance, the intrinsic fluorescence intensity of a CFP-C3G mixture (32.81 × 10^−6^ mol/L of C3G) incubated at 298 K, 304 K, and 310 K was quenched to 2460.67 a.u., 2422.93 a.u., and 2398.67 a.u., respectively. Similar results were found in the interaction with other proteins, such as β-conglycinin [21], and β-casein [22]. However, the maximum fluorescence emission wavelength of β-conglycinin did not shift significantly in the presence of C3G, while that of β-casein showed an approximately 5 nm blue shift. This highlights that the extent of C3G-induced protein conformational changes is protein-specific.

### 3.2. Fluorescence Quenching Mechanism

To explore the interaction mechanism between C3G and CFP, the Stern-Volmer equation (Equation (3)) was used to fit the fluorescence quenching data:

(3) $$\frac{F_{0}}{F}=1+K_{Q}\;\tau_{0}\;[Q]=1+K_{\mathrm{sv}}\;[Q]$$

Here, F_0_ represents the initial fluorescence intensity of CFP, F is the fluorescence intensity of CFP in the presence of C3G, [Q] is the molar concentration of C3G, K_Q_ represents the biomolecular quenching rate, K_sv_ is the Stern-Volmer quenching constant, and τ_0_ (τ_0_ = 10^−8^ s) is the average lifetime of the fluorescent molecule in the absence of the quencher (C3G).

The linear Stern-Volmer plots of F0/F against [Q] at different temperatures (298, 304, and 310 K) are shown in Figure 1(B-1), and the relative parameters are summarized in Table 1. Obviously, the correlation coefficients are all higher than 0.99, indicating good linearity and confirming that the data could be used to analyze the quenching mechanism [23]. Fluorescence quenching can be classified into dynamic and static quenching mechanisms based on the quench process. In general, static quenching is caused by the formation of a non-fluorescence complex between the quencher and the fluorophore [24]. As a result, the quenching constant (K_sv_) decreases with increasing temperature due to the dissociation of weak non-covalent bonds [25]. In contrast, dynamic quenching results from collision among particles. Higher temperatures may enhance diffusion, resulting in greater fluorescence and increased K_sv_ values [26]. As shown in Table 1, the values of K_sv_ at different temperatures (298, 304, and 310 K) were 1.89 × 10^4^ L∙mol^−1^, 1.80 × 10^4^ L∙mol^−1^, and 1.07 × 10^4^ L∙mol^−1^, respectively, suggesting that quenching may be initiated by static quenching. Additionally, the K_Q_ value is a critical parameter for distinguishing quenching types: if K_Q_ far exceeds the maximum scattering collision quenching constant (2.0 × 10^10^ L·mol^−1^·s^−1^) between the quencher and biopolymers, static quenching is confirmed [27]. It is apparent that K_Q_ in the quenching process at three temperatures was around 10^12^, confirming that the bonding of C3G to CFP belongs to a typical static quenching mechanism.

For static quenching, a double logarithmic equation (Equation (4)) can be used to calculate the binding constant (K_a_) and the number of binding sites (n):

(4) $$\log\;\frac{F_{0}\;-\;F}{F}\;=\;log\;K_{a}\;+\;n\;log\;[Q]$$

As demonstrated in Table 2, the number of binding sites (n) for the CFP-C3G interaction was 1.07 at 298 K, 1.29 at 304 K, and 1.41 at 310 K. This indicates that CFP possesses approximately one specific binding site for C3G [13], which is consistent with findings for the C3G-ovalbumin system [25]. However, a key difference emerges in the ovalbumin system, K_a_ decreased with increasing temperature, whereas in our study, K_a_ increased (3.72 × 10^4^ L·mol^−1^ at 298 K vs. 62.55 × 10^4^ L·mol^−1^ at 310 K). This increase suggests that the CFP-C3G binding interaction is endothermic, and elevated temperatures promote complex formation. The discrepancy in K_a_ trends may stem from differences in protein amino acid composition: ovalbumin contains more polar residues (e.g., Asp, Glu) that form hydrogen bonds with C3G (an exothermic interaction), while CFP has more hydrophobic residues (e.g., Leu, Ile) that drive binding via hydrophobic interactions (endothermic, as confirmed in Section 3.3).

Notably, the trend of K_a_ (increasing with temperature) contrasts with that of K_sv_ (decreasing with temperature). This inconsistency implies a dual effect of temperature on the CFP-C3G system: higher temperatures promote complex formation (increasing K_a_) but simultaneously dissociate weakly bound complexes (decreasing K_sv_). This suggests the potential coexistence of dynamic quenching, albeit secondary to static quenching. This phenomenon is similar to the C3G-α-lactalbumin system [28], where static quenching dominated but dynamic quenching was also detected, highlighting that polyphenol–protein interactions often involve mixed quenching mechanisms, with the dominant type depending on protein structure and environmental conditions.

### 3.3. Thermodynamic Analysis

In order to further study the thermodynamic parameters involved in the interaction between CFP and C3G, the Van’t Hoff equation was introduced to calculate the enthalpy change (∆H), entropy change (∆S), and free energy change (∆G). These are commonly defined as follows: ΔH quantifies the heat exchanged, where a positive value indicates an endothermic process, and a negative value an exothermic one. ΔS reflects the change in system disorder, with a positive ΔS denoting an increase in randomness. Ultimately, ΔG, which is determined by both ΔH and ΔS, governs the reaction spontaneity, with a negative ΔG signifying a spontaneous process.

(5) $$In\;K_{a}\;=\;-\;\frac{∆R}{\mathrm{RT}}\;+\;\frac{∆S}{R}$$

∆G = ∆H − T∆S(6)

Here, R (8.314 J∙mol^−1^∙K^−1^) is the gas constant, T is the absolute temperature, and K_a_ is the binding constant.

Thermodynamic parameters are instrumental in elucidating the interaction forces (hydrogen bonds, hydrophobic forces, and electrostatic forces) between polyphenols and proteins [7]. In general, hydrophobic interactions are typically endothermic, as energy is required to break the hydration shells around non-polar groups. In contrast, the formation of hydrogen bonds or electrostatic interactions is generally exothermic. As documented in the literature, the nature of intermolecular forces can be comprehensively analyzed through the values of ∆H and ∆S [29]. Specifically, when ∆H > 0 and ∆S > 0, hydrophobic forces are predominant; when ∆H < 0 and ∆S < 0, van der Waals forces and hydrogen bonds are the primary interactions; when ∆H < 0 and ∆S > 0, electrostatic interactions are the main forces; and when ∆H > 0 and ∆S < 0, both electrostatic and hydrophobic forces drive the interaction [30].

**Figure 2:**
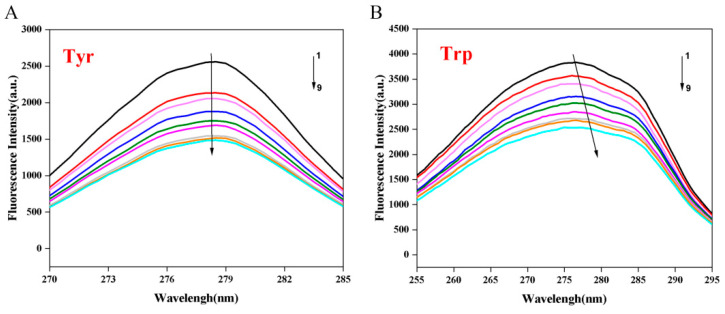
Synchronous fluorescence spectra of the interaction between C3G and CFP (C3G concentration ranges from 0 to 35.87 × 10^−6^mol/L). (**A**) Spectrum for tyrosine (Tyr); (**B**) Spectrum for tryptophan (Trp). 1: low C3G concentration; 9: high C3G concentration.

**Figure 3:**
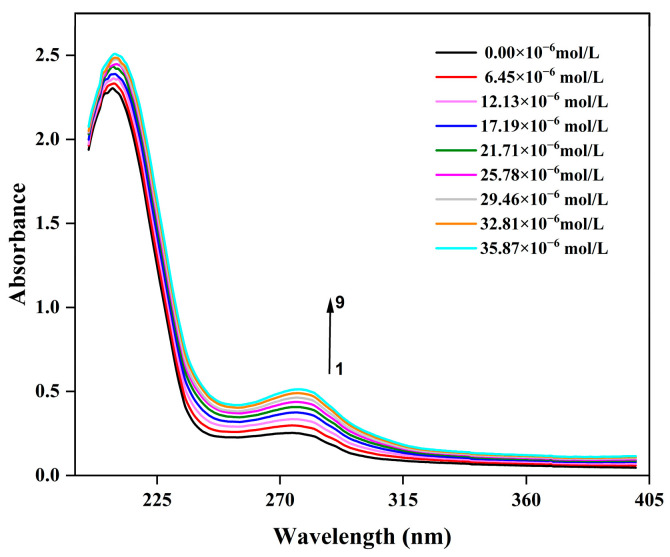
UV-vis absorption spectra of the interaction between C3G and CFP.

Within this section, the Van’t Hoff plot (Figure 1(B-3)) was obtained by fitting the relevant experimental data to Equation (5). Subsequently, the values of ∆G, ∆H, and ∆S were calculated using Equation (6), with the results presented in Table 2. Notably, both ∆H (181.45 kJ∙mol^−1^) and ∆S (0.70 kJ∙mol^−1^) were positive, suggesting that hydrophobic interactions predominantly govern the non-covalent binding of C3G and CFP. Positive ΔS also indicates an increase in system disorder, which may result from the release of water molecules from the hydration layers during the interaction between the hydrophobic residues of CFP and C3G’s anthocyanin core. In addition, the ∆G was negative and became more negative (−26.09 kJ∙mol^−1^∙K^−1^ at 298 K, −34.41 kJ∙mol^−1^∙K^−1^ at 310 K) as the temperature increased, suggesting that the binding process between CFP and C3G is spontaneous and higher temperatures enhance the spontaneity of complex formation, which is consistent with the endothermic nature of hydrophobic interactions. Furthermore, the positive value of ∆H further confirms that the binding interaction between the molecules was endothermic. This helps explain the decreased fluorescence intensity with temperature, as stated in Section 3.1, since elevated temperature promotes the formation of new and boosts the collision between them, both of which induce diminished fluorescence. These findings align with previous observations of interactions between C3G and various proteins, such as α-lactalbumin and β-casein [28,31], where hydrophobic forces also dominated. However, the enthalpy change (ΔH) for the CFP-C3G interaction was determined to be 181.45 kJ·mol^−1^, which is notably higher than the 25 kJ·mol^−1^ reported for the α-lactalbumin system. This substantial disparity may originate from the distinct amino acid composition of CFP. As quantified in our previous study [12], CFP possesses a higher proportion of hydrophobic residues (35%), which may promote stronger hydrophobic interactions with C3G.

### 3.4. Synchronous Fluorescence Spectroscopy

Synchronous fluorescence spectroscopy, a technique extensively employed to investigate the microenvironment of luminescent amino acid residues in proteins, is particularly useful for examining the conformational changes in proteins upon binding with polyphenols [32]. Figure 2 presents the synchronous fluorescence spectra of CFP solutions at varying concentrations of C3G. Notably, the fluorescence intensity of Trp was higher than that of Tyr in CFP, which suggests that Trp is indeed the primary contributor to the fluorescence signal of CFP. However, in the presence of C3G, the fluorescence intensity significantly decreased, and when the concentration of C3G reached 35.87 × 10^−6^ mol/L, there was a decrease of approximately 40.96% in Tyr fluorescence intensity, which was significantly greater than that in Trp (33.79%). This observation suggests that Tyr may play a more prominent role in the binding process than Trp and is likely situated closer to the binding site [13]. Furthermore, the maximum peak positions of Tyr and Trp did not exhibit significant changes, suggesting that the microenvironments of these amino acids were not substantially affected by C3G. This finding contrasts with the observations reported by Wang et al. [12], who noted a 2 nm upward shift in the maximum absorption wavelength of Trp in the presence of chlorogenic acid, which altered the local environment of Trp and increased the exposure of Trp residues to the hydrophilic phase. These discrepancies may arise from differences in molecular structure, which subsequently influence their interactions with CFP. C3G, as an anthocyanin glycoside, comprises a hydrophobic anthocyanin core (polycyclic aromatic hydrocarbons) and a hydrophilic glucose moiety, thereby exhibiting amphiphilic properties, although its primary mode of binding to proteins was through hydrophobic interactions (as deduced in Section 3.3). Moreover, the steric hindrance and hydrophilicity of its glucose group likely impede significant perturbation of the Trp microenvironment’s polarity, thus not markedly altering the fluorescence spectrum of Trp. In contrast, chlorogenic acid, as the ester formed between caffeic acid and quinic acid, has multiple phenolic hydroxyl and carboxylic acid groups within its molecular structure, resulting in strong polarity. When encountering proteins, it may also interact with polar residues surrounding Trp through hydrogen bonding or electrostatic interactions, although hydrophobic interactions are the main forces. This interaction might alter the local polarity around Trp, leading to a red shift in its fluorescence spectra and an increase in microenvironmental polarity. 

### 3.5. UV-Vis Absorption Spectral Analysis

Owing to its high sensitivity to changes in the microenvironment of molecular chromophores, ultraviolet-visible (UV-Vis) absorption spectroscopy has become a classic and efficient technical tool for studying the interactions between proteins and small molecules (such as polyphenols, drug molecules, and metal ions), as well as identifying the formation of complexes [33]. As can be seen in Figure 3, the system exhibits two absorption peaks near 210 nm and 280 nm, respectively. These were ascribed to the characteristic absorption peaks of polypeptide backbone and aromatic amino acid residues (Trp and Try), respectively [34]. Figure 3 illustrates that as the concentration of C3G increased from 6.45 × 10^−6^ mol/L to 35.87 × 10^−6^ mol/L, the absorbance of CFP gradually rose. This observation indicates that C3G has interacted with the aromatic amino acid residues of CFP, thereby forming a new type of complex. Notably, there is no significant shift in the peak positions, indicating that the hydrophobicity of the microenvironment surrounding the aromatic amino acid residues remained unchanged [35]. Furthermore, UV-vis absorption spectroscopy can also be used to differentiate quenching mechanisms. Dynamic quenching affects only the excited state of the quenching molecules without altering the absorption spectrum of the quenching substances, whereas static quenching impacts the absorption spectrum [36]. Consequently, it might be concluded that dynamic quenching may also occur in the CFP-C3G system, aligning with the conclusions drawn from the aforementioned fluorescence data. These findings are consistent with results obtained in the C3G and ovalbumin system, where static quenching was predominant, accompanied by dynamic quenching [25].

### 3.6. Particle Size and Zeta Potential

**Figure 4:**
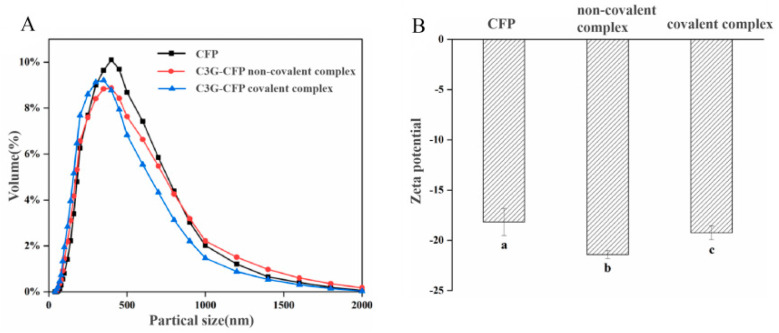
Changes in particle size distribution (**A**) and zeta potential (**B**) before and after binding of C3G to CFP. Different lowercase letters (a, b, c) in (**B**) indicate significant differences (*p* < 0.05) among the groups.

Figure 4A presents the particle size distribution and surface charge of CFP before and after binding to C3G. It is obvious that the average particle size of CFP was 425.1 nm, whereas after non-covalently and covalently binding with C3G, it decreased to 400.8 nm and 388.9 nm, respectively. Notably, the maximum peaks shifted to smaller sizes and the particle size distribution became narrower, indicating that the introduction of C3G promotes the formation of a more compact structure in CFP.

Figure 4B illustrates the variation in the surface charge of CFP in the presence of C3G. Initially, CFP existed as negatively charged nano-colloidal particles (−18.2 mV). Upon interaction with C3G, both non-covalent and covalent interactions contributed to an increase in the negative surface charge (−25.6 mV and −22.1 mV, respectively), with the enhancement in the non-covalent component being more pronounced. According to the literature, protein-based colloidal particles exhibit high sensitivity to environmental factors such as ligand presence, pH, and ionic strength, all of which can influence surface charge and, consequently, the dispersion stability of the particles [32]. The observed increase in the absolute ζ-potential value within these composite systems can be attributed to C3G, which may act as a negatively charged ligand, interacting with CFP and augmenting the surface charge of the particles. The formation of covalent bonds could consume certain polar groups of C3G (e.g., hydroxyl groups), leading to a greater reduction in the total number of charged groups on the complex surface. From an application perspective, the more negative zeta potential of non-covalent complexes may enhance their dispersion stability in aqueous food systems (e.g., beverages) by reducing sedimentation during storage. In addition, this interaction might also account for the observed decrease in average particle size. The increase in the absolute ζ-potential value may provide an electrical repulsion force, which may prevent particle aggregation and promote greater separation from each other through stronger steric hindrance and electrostatic repulsion, resulting in smaller particle size [21].

However, these findings are not entirely consistent with our previous study on the interaction between chlorogenic acid and CFP [12], which demonstrated that covalent binding with chlorogenic acid led to a more compact structure, while non-covalent interactions resulted in a looser structure. This discrepancy may be due to structural differences between C3G and chlorogenic acid. C3G, as an anthocyanin glycoside, comprises a hydrophobic anthocyanin core (polycyclic aromatic hydrocarbons) and a hydrophilic glucose moiety, resulting in greater hydrophobicity compared to chlorogenic acid. This characteristic facilitates C3G’s participation in hydrophobic interactions, which predominantly govern the non-covalent binding, leading to the formation of compact complexes. In conclusion, interaction occurred between C3G and CFP, resulting in the formation of a stable complex and alteration in the particle size and zeta potential of CFP.

### 3.7. Antioxidant Properties Determination

#### 3.7.1. ABTS Radical Scavenging Activity

**Figure 5:**
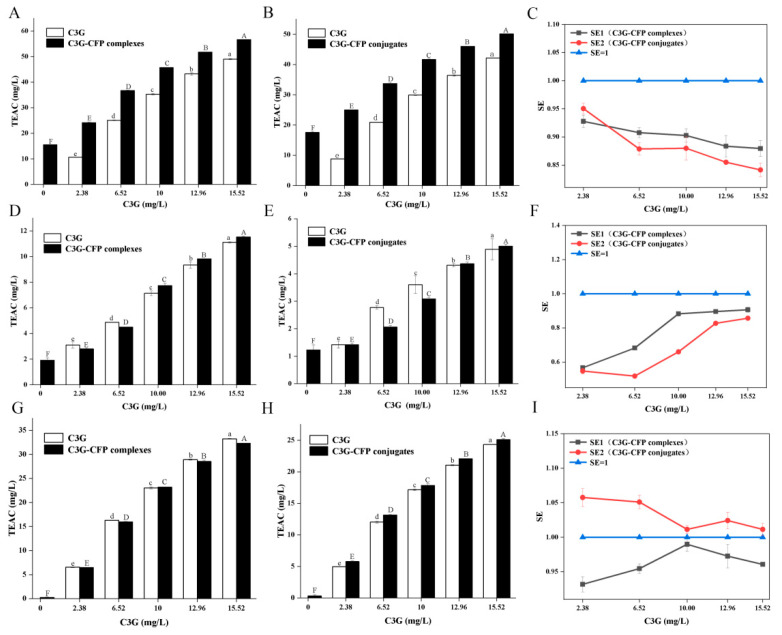
Antioxidant activities and synergistic effects of C3G-CFP complexes assessed via ABTS (**A**–**C**), DPPH (**D**–**F**), and FRAP (**G**–**I**) assay. The mean values for each sample with different uppercase and lowercase letters are significantly different (*p* < 0.05).

The ability of free C3G, CFP, C3G-CFP non-covalent complexes, and C3G-CFP conjugates to scavenge ABTS free radicals was determined by the ABTS method, and the experimental results are shown in Figure 5A. Obviously, the ABTS scavenging activity of CFP alone was very low, while after physically or chemically reacting with C3G, the activity significantly increased. Moreover, the scavenging capacity of both the C3G-CFP complex and conjugate exhibited a concentration-dependent relationship with C3G. Compared to the C3G-CFP conjugate, the antioxidant capacity of the C3G-CFP complex was higher. For example, the scavenging activity of CFP alone was lower than 20 mg/L TEAC, while in the presence of 49.05 mg/L of C3G, those of the C3G-CFP complex and C3G-CFP conjugate increased to 56.63 mg/L and 50.09 mg/L TEAC, respectively. This phenomenon suggests that the induced C3G exerted a positive effect on CFP to scavenge ABTS radical. As documented in the literature, the ABTS assay is a commonly used spectrophotometric method to assess the antioxidant activity of compounds [37]. The method is based on the hydrogen donation capability of antioxidants to reverse ABTS∙^+^ radical cations to ABTS. Furthermore, C3G is an excellent antioxidant with five phenolic hydroxyl groups in its molecule. Consequently, the increased scavenging effect of CFP on ABTS radicals may be due to the bonded C3G, which may introduce certain free phenolic hydroxyl groups into CFP molecule through non-covalent or covalent interactions and enhanced the antioxidant activity of CFP in the end. Although certain hydroxyl groups in C3G participate in the interaction with CFP, the residue groups could still act as hydrogen donors to reverse ABTS^+^·and enhance the antioxidant activity of CFP. Meanwhile, the lower scavenging activity of conjugates compared to the non-covalent ones may be due to the stronger interaction forces, which may confine the freedom of hydroxyl groups in C3G.

In addition, it is important to note that CFP itself exhibits certain antioxidant activities, potentially due to the presence of specific hydrogen donor residues, such as tryptophan, phenylalanine, histidine, tyrosine, methionine, cysteine, and proline, among others. [12]. As discussed in Section 3.1, C3G may alter the conformation of CFP, which might increase the exposure of these endogenous antioxidant groups, thereby enhancing the radical scavenging capacity of CFP. Furthermore, to accurately assess the impact of C3G interaction on the antioxidant activity of CFP, the pH conditions for CFP and free C3G were kept consistent with the respective reaction conditions (the pH values for the C3G-CFP complex and C3G-CFP conjugate were 7.0 and 9.0, respectively). The data indicate that the solvent environment significantly influenced the antioxidant activity [38]. For instance, the scavenging activity values of CFP alone and free C3G (15.52 mg/L) at pH 7.0 were 15.50 mg/L and 15.52 mg/L, respectively, whereas they were 17.55 mg/L TE and 42.14 mg/L at pH 9.0, respectively. In this sense, it could be deduced that an alkaline environment may be more suitable for compounds to exhibit antioxidant activity. Therefore, the C3G-CFP conjugate should exhibit higher ABTS radical scavenging activity if its antioxidant is equal to that of the C3G-CFP complex. Although alkaline conditions may be expected to enhance the scavenging ability of the C3G-CFP conjugate, the opposite was observed. The antioxidant of the C3G-CFP non-covalent complex was higher than that of the conjugate, which further implies the beneficial effects of non-covalent interactions on improving the antioxidant capacity of CFP.

In order to further explore the synergistic effect between C3G and CFP, an in-depth analysis of the experimental data was carried out and the results are shown in Figure 5C. Obviously, the SE of all samples was lower than 1, and increased C3G concentrations induced diminished values. In general, when SE > 1, it indicates that there is a synergistic effect between the two compounds; when SE < 1, it shows an antagonism effect; when SE = 1, it represents an additive effect. In this sense, antagonism phenomena occurred when C3G interacted with CFP either through non-covalent or covalent interaction. The antagonism effect among antioxidants is based on the regeneration of less effective antioxidants by more effective antioxidants [39]. Here, C3G exerted stronger antioxidant capacity than CFP and acted as the dominant antioxidant as the result of its stronger hydrogen donor abilities. Consequently, the formation and regeneration of antioxidant radical adducts may co-exist, along with the alterations in the CFP microenvironment caused by C3G in their complexes. On one hand, the freedom of hydrogen in C3G is confined to a certain extent because of the interaction, leading to decreased hydrogen donor abilities. On the other hand, the interaction may also induce the exposure of some endogenous antioxidant groups, resulting in enhanced antioxidant capacity. As a result, the competition between these two factors may affect the overall antioxidant activity of CFP.

#### 3.7.2. DPPH Radical Scavenging Capacity

DPPH is a stable free radical characterized by its deep purple methanolic solution, which exhibits strong absorbance at 517 nm. Upon the introduction of a substance with antioxidant properties, its hydrogen atoms are donated, converting the DPPH radical into its non-radical form, DPPH-H [40]. This reaction results in a lighter solution color and a decrease in absorbance at 517 nm. Consequently, the extent of absorbance reduction serves as an indicator of the antioxidant’s efficacy in scavenging DPPH radicals; a more significant decrease in absorbance correlates with a stronger scavenging capability.

Figure 5D provides specific data illustrating that CFP alone demonstrated a limited capacity to scavenge DPPH radicals. However, upon complexation with C3G to form the C3G-CFP complex, there is a marked enhancement in its radical scavenging ability. With the increase in the concentration of C3G, the scavenging efficacy of the C3G-CFP complex also significantly improved (*p* < 0.05).

A similar trend was observed in the conjugates, but their antioxidant capacity was markedly lower than that of the complexes (Figure 5E). These findings are consistent with those obtained from the ABTS assay, further supporting the hypothesis that the combination of C3G and CFP enhances the antioxidant performance of CFP. However, when compared to the ABTS assay data, it is evident that the antioxidant activity of the same samples was greatly lower in the DPPH assay. For example, the antioxidant capacity of CFP measured in the ABTS assay was substantially higher than 10 mg/mL, whereas it was lower than 3 mg/mL in the DPPH assay. These differences may be attributed to the distinct antioxidant mechanisms involved [12]. Furthermore, Figure 5F demonstrates that C3G exhibited an antagonistic effect with CFP, consistent with the ABTS assay results. However, the SE values increased with higher C3G concentrations, and the values for the complexes were greater than those for the conjugate. The increased SE suggests that the antagonistic effect gradually diminished at higher C3G concentrations. In our experiment, the concentration of CFP was kept constant, indicating that the binding sites were limited. When the concentration of C3G is relatively high, leading to the saturation of binding sites in CFP, the number of C3G molecules that interact with CFP may decrease, thereby enhancing the hydrogen donor ability of the system. The lower SE for the conjugate may be attributed to the stronger interaction forces, which restrict the mobility of hydroxyl groups in C3G. Furthermore, the steric accessibility of the antioxidant to the radical site in DPPH· may also play a significant role in radical scavenging [41]. As discussed in Section 3.6, the generated C3G-CFP conjugates are more tightly bound than the non-covalent complex. The steric hindrance caused by the compact structure may partially impede the phenolic OH groups from reaching the radical site, resulting in decreased antioxidant capacity. This indicates that the strength of the synergistic effect between C3G and CFP depends not only on their binding type but also on the concentration of C3G.

#### 3.7.3. Reducing Power of C3G-CFP

Reducing power serves as an important indicator of antioxidant capacity, based on the ability of antioxidants to reduce Fe^2+^. In this section, the antioxidant activity of CFP, both before and after covalent and non-covalent bonding with C3G, is assessed using the FRAP method. As can be seen in Figure 5G,H, CFP exhibited relatively weak reducing capacity when present alone. However, upon binding with C3G, whether through covalent or non-covalent interactions, its reducing ability was significantly enhanced (*p* < 0.05). Furthermore, the reducing power increased proportionally with higher concentrations of C3G. These findings are consistent with the results obtained from the DPPH and ABTS assays. Notably, although the concentrations of CFP and C3G across these assays were the same, the antioxidant capacities of the samples varied. For example, the antioxidant capacity of CFP alone was 15.50 mg/mL of TEAR in the ABTS method, whereas it was only 1.91 and 0.25 mg/mL of TE in the DPPH and FRAP methods, respectively. Similar results were also found for the free C3G and CFP-C3G complexes, suggesting that one antioxidant may exert various antioxidant capacities across different systems.

The synergistic effect data indicate that the SE values were all lower than 1 in the non-covalent system, suggesting that the non-covalent binding of C3G exhibited an antagonistic effect with CFP. In contrast, within the covalent system, the SE values ranged from 1 to 1.07, indicating that this type of combination demonstrates a synergistic effect on reducing Fe^3+^-TPTZ. Furthermore, as the concentration of C3G increased, the SE of complexes initially increased (2.38–10.00 mg/mL) and then decreased (>10.00 mg/mL). However, a significant decreasing trend was observed for the conjugate in the range of 2.38 to 10.00 mg/mL, followed by a decrease with some fluctuation thereafter. Consequently, the synergistic effect was largely dependent on both the concentration of C3G and interaction types. As documented, an SE value greater than 1 indicates the formation of more potent antioxidant intermolecular complexes, whereas an SE value less than 1 implies that a more effective antioxidant generates a less effective one [12]. The observed variations in the SE of complexes and conjugates as a function of C3G concentrations suggest that the addition of 2.38 mg/mL and 10.00 mg/mL of C3G, respectively, may be better for these two types of complexes to exhibit higher synergistic antioxidant effects.

## 4. Conclusions

In this study, two primary experiments were conducted. Firstly, the potential interaction mechanism underlying the physical mixture of C3G and CFP was investigated. The results indicate that C3G spontaneously bonded to CFP at a molar ratio of 1:1, resulting in static quenching accompanied by dynamic quenching of the intrinsic fluorescence of CFP, increased polarity around the Trp residue microenvironment, and the formation of non-covalent complexes with a compact structure. Furthermore, the binding was identified as an endothermic reaction primarily driven by hydrophobic interactions. The steric hindrance and hydrophilicity of the glucose group in C3G may be due to these phenomena. Secondly, the impact of non-covalent bonding on the antioxidant activity and particle properties of CFP was examined in comparison to C3G-CFP conjugates. The findings reveal that although a single antioxidant exhibited varying antioxidant capacities across different assays, the formed non-covalent complex demonstrated a stronger antioxidant capacity than the conjugate and CFP alone, with further enhancement observed as the concentration of C3G increased. Nevertheless, no synergistic effects were detected when combining C3G and CFP using the ABTS and DPPH assays. Conversely, in the FRAP assay, the covalent C3G-CFP complex demonstrated a slight synergistic effect (SE > 1) at lower C3G concentrations. However, more studies are still needed to identify the precise binding sites between C3G and CFP, and to reveal the specific mechanism by which their interaction regulates antioxidant properties.

## Figures and Tables

**Table 1:** K_sv_, n, K_a_, and R of the interaction between C3G and CFP.

Complexes	T(K)	K_sv_ (×10^4^ L∙ mol^−1^)	K_Q_ (×10^12^ L∙ mol^−1^∙S^−1^)	R_a_^2^	K_a_ (×10^4^ L∙mol^−1^)	n	R_b_^2^
C3G-CFP	298	1.89 ± 0.07 ^A^	1.89 ± 0.07 ^A^	0.99	3.72 ^C^	1.07 ± 0.04 ^C^	0.99
	304	1.80 ± 0.12 ^B^	1.80 ± 0.12 ^B^	0.97	31.21 ^B^	1.29 ± 0.05 ^B^	0.99
	310	1.07 ± 0.06 ^C^	1.07 ± 0.06 ^C^	0.98	62.55 ^A^	1.41 ± 0.03 ^A^	0.99

Note: Data with different letters in the same column indicate significant differences (*p* < 0.05). R_a_ is the correlation coefficient for the K_sv_ values. R_b_ is the correlation coefficient for the K_a_ values.

**Table 2:** Thermodynamic parameters.

Complexes	T(K)	∆G (kJ∙mol^−1^)	∆S (kJ∙mol^−1^∙K^−1^)	∆H (kJ∙mol^−1^)
C3G-CFP	298	−26.09	0.70	181.45
	304	−31.99		
	310	−34.41		

## Data Availability

The original contributions presented in this study are included in the article. Further inquiries can be directed to the corresponding author.

## Abbreviations

The following abbreviations are used in this manuscript:

CFP	Corbicula fluminea protein
C3G	Cyanidin-3-O-glucoside
UV-Vis	Ultraviolet-visible
SFS	Synchronous fluorescence spectroscopy
ABTS	2,2′-Azinobis-(3-ethylbenzothiazoline-6-sulfonate)
DPPH	1,1-diphenyl-2-picrylhydrazyl
FRAP	Ferric ion-reducing antioxidant power
TPTZ	2,4,6-Tri(2-pyridyl)-s-triazine
Trp	Tryptophan
Tyr	Tyrosine
TEAC	Trolox equivalent antioxidant capacity
